# Histological Changes in Gills of Two Fish Species as Indicators of Water Quality in Jansen Lagoon (São Luís, Maranhão State, Brazil)

**DOI:** 10.3390/ijerph111212927

**Published:** 2014-12-12

**Authors:** Débora M. S. Santos, Mércia Regina S. Melo, Denise Carla S. Mendes, Iolanda Karoline B. S. Rocha, Jakeline Priscila L. Silva, Sildiane M. Cantanhêde, Paulo C. Meletti

**Affiliations:** 1 Departamento de Química e Biologia, Universidade Estadual do Maranhão, São Luís—MA, 65055-000, Brazil; E-Mails: mercia21melo@gmail.com (M.R.S.M.); denisemendes2@yahoo.com.br (D.C.S.M.); iolanda.rbarros@gmail.com (I.K.B.S.R.); jakelinepriscila@gmail.com (J.P.L.S.); sildianebio@gmail.com (S.M.C.); 2 University City Paulo VI, s/n^o^, Cidade Operária, São Luís—MA, 65055-000, Brazil; 3 Departamento de Ciências Fisiológicas, Universidade Estadual de Londrina, PR, 86057-970, Brazil; E-Mail: pcmeletti@gmail.com (P.C.M.)

**Keywords:** *Centropomus undecimalis*, *Sardinella* sp., lagoon environment, histological biomarkers

## Abstract

Water quality of the Jansen Lagoon (São Luís, Maranhão State, Brazil) was assessed through histological biomarkers and microbiological parameters. To this end, 29 fish specimens (11 *Centropomus undecimalis* and 18 *Sardinella* sp) and eight water samples were collected during the rainy and dry periods of 2013. The lagoon water showed thermotolerant coliform indices above the limit set forth in CONAMA Resolution 357/2005. Histological changes observed in the gills were: lifting of the respiratory epithelium, hyperplasia of the lamellar epithelium, incomplete and complete fusion of several lamellae, disorganization of the lamellae, congestion of blood vessels, aneurysms, hypertrophy of the respiratory epithelium, hemorrhage and rupture of the lamellar epithelium and parasite. The histological alteration index (HAI) average value to *Sardinella* sp was 31.8 and to *C. undecimalis* was 22.2. The average HAI value in both species corresponds to category 21–50, with tissue injuries being classified from moderate to severe. The presence of histological injuries and the HAI values indicate that the fish sampled from the Jansen Lagoon are reacting to non-specific xenobiotics present at the site.

## 1. Introduction

Aquatic ecosystems are exposed to excessive input of pollutants and contaminants from various sources like domestic and industrial sewage, agricultural processes, heavy metals and others. The uncontrolled discharge of these compounds into the water directly affects aquatic organisms, including fish, which are considered a bioindicator of environmental pollution or contamination [1]. Bioindicators are sentinel species used as primary indicators of abiotic or biotic changes presented by a certain environment in the presence of pollutants [2]. Changes in organs of fish, such as the gills, are good biomarkers of water contamination. Biomarkers are biological indicators showing the effects resulting from exposure to a stressor, and can be identified by biochemical, cellular, histological and behavioral changes [3]. Gills of fish are extremely sensitive to chemical and physical modifications in the environment, mainly because of the large surface of the respiratory epithelium and the high perfusion rate that facilitate the entry of pollutants into this tissue [4,5,6,7] In this way, morphological changes in the gills are widely used as parameters in biomonitoring programs, for they are defense mechanisms to potential stressors of the aquatic environment [8]. Coliforms are also used as indicators of contamination and have been used since the nineteenth century as a basic bacteriological parameter in the definition of standards for the characterization and assessment of water quality in general. Bacteria of this group are the most frequently used as indicators of contamination, because they point out the possibility of the presence of fecal pollution, indicating that the water body receives sewage, directly or indirectly [9]. Given the above, this study evaluated the water quality of the Jansen Lagoon using microbiological parameters and histological lesions in the gills of two fish species (*Centropomus undecimalis* and *ardinella* sp.) as biomarkers.

## 2. Experimental Section

### 2.1. Study Area

**Figure 1:**
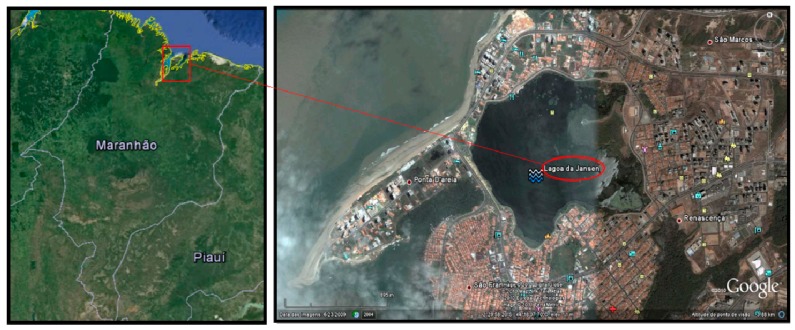
Lagoon Jansen, São Luís, Maranhão, Brazil. Source: Google Earth.

Jansen Lagoon is located on the west side of São Luís Island, Maranhão State, Brazil, between the coordinates 2°29ʹ07ʹʹ S and 44°18ʹ02ʹʹ W within a metropolitan area of high real estate value (Figure 1). It is an environment of anthropogenic origin, created in the 70 s, due to the damming of two streams, Ana Jansen and Jaracati, for the construction of avenues for access to adjacent neighborhoods. Urbanization in the surroundings by residences, buildings and restaurants has contributed to the discharge of domestic sewage, which impairs water quality and may cause water borne diseases, since the fishery resources (fish, crustaceans and mollusks) have been used by the riverine population for subsistence and marketing.

### 2.2. Fish Sampling

A total of 29 fish specimens was collected in the rainy and dry periods of 2013, at a site located at the coordinates 2°29ʹ40ʹʹ S and 44°18ʹ13ʹʹ W, in the Jansen Lagoon, these being 11 *C. undecimalis* (common name: Camorim) in the rainy period of 2013 and 18 *Sardinella* sp (common name: sardine), five in the rainy and 13 in the dry periods of 2013. The fish were captured by artisanal fisheries using seining nets. After collection, fish were taken in plastic boxes with water from the site itself to the Laboratory of Animal Morphophysiology, Department of Chemistry and Biology, State University of Maranhão—Brazil, where they were euthanized by thermal shock. To verify the reversibility of the lesions, three camorim samples were used as control; they were acclimatized in a 50 L aquarium with constant aeration, light/dark 12 h/12 h photoperiod and temperature of 29 ºC for 30 days, initially with water from the collection site itself. During the acclimatization, the water was gradually replaced by artesian well water. Only one fish survived.

### 2.3. Histopathological Analysis

In the laboratory, the second right gill arch was removed, fixed in 10% formalin for 24 h and then decalcified in 10% nitric acid, dehydrated in increasing concentrations of alcohols, cleared in xylene, impregnated and embedded in paraffin. Five μm thick sections were stained with hematoxylin and eosin for histological description [10]. Gill histological changes were evaluated semi-quantitatively by calculating the Histological Alteration Index (HAI), based on the severity of each lesion [5]. The changes were classified into progressive stages of tissue damage: stage I, which do not affect the functioning of the organ; stage II, more severe and impair the normal functioning of the organ; and stage III, very severe and irreversible. For each fish, HAI was calculated using the formula: HAI = 1 × ∑ *I* + 10 × ∑ *II* + 100 × ∑ *III*, with *I*, *II* and *III* corresponding to the number of alterations of stage I, II and III. The average HAI was divided into five categories: 0–10 = normal functioning of the tissue; 11–20 = mild to moderate alteration; 21–50 = moderate to severe alteration; 51–100 = severe alteration; ≥100 irreparable alteration.

### 2.4. Microbiological Analysis of Water

For bacteriological analysis of water from the Jansen Lagoon, eight samples were collected during the rainy and dry periods of 2013. For the collection, sterilized vials (500 mL) were used and samples were obtained at a depth of 80 cm below the surface, then packaged in isothermal material containing ice cubes and transported to the Laboratory of Water and Food Microbiology, Course of Veterinary Medicine, State University of Maranhão‒Brazil. In the analysis, we determined the most probable number (MPN) of thermotolerant coliforms using the multiple tube technique with a series of five tubes [11]. 

## 3. Results and Discussion

### 3.1. Assessment of Microbiological Parameters of the Lagoon Water

Microbiological parameters (thermotolerant coliforms) of the Jansen Lagoon were evaluated in two collection periods. The first, during the rainy period, and the second, during the dry period of 2013. Values obtained are listed in Table 1.

**Table 1:** Most Probable Number (MPN) of thermotolerant coliforms per 100 mL of water samples from the Jansen Lagoon, during the dry and rainy periods of 2013, São Luís, Maranhão State, Brazil.

Collection/Period	Samples	Thermotolerant Coliforms
1st/Dry	2	≥1600/mL
2nd/Dry	2	≥1600/mL
3rd/Rainy	2	≥1600/mL
4th/Rainy	2	≥1600/mL

The CONAMA Resolution 357 of 17 March 2005 [12], mentions that a brackish water body intended to protect aquatic communities (class 1), shall not exceed a limit of 1000 fecal coliforms per 100 mL in 80% of samples. In 2011, the Environmental Department of the State of Maranhão [13], found 78 points of direct discharge of sewage into the lagoon, a fact that contributes to outcomes achieved in the research that showed values of thermotolerant coliforms above those permitted by the resolution mentioned.

Rojas *et al.*, [14] analyzed physico-chemical and nutritional characteristics of the Jansen Lagoon water. According to their results, the high values of some nutrients such as phosphate, nitrite, and sulfide classify the lagoon as a eutrophic and contaminated environment. The authors affirm that the excess of these nutrients come from the urban sewage system.

The presence of the Coliform bacteria group has a direct relationship to fecal contamination, as they inhabit the gastrointestinal tract of human beings and other homeothermic animals [15,16]. Research conducted by Cantanhêde *et al.*, [17] where assessed the lagoon water and verified high levels of total and thermotolerant coliforms in the samples analyzed, the results suggest that the Jansen Lagoon is unfit for the purpose of water recreation and use of its fishing resources.

### 3.2. Histopathological Evaluation

**Figure 2:**
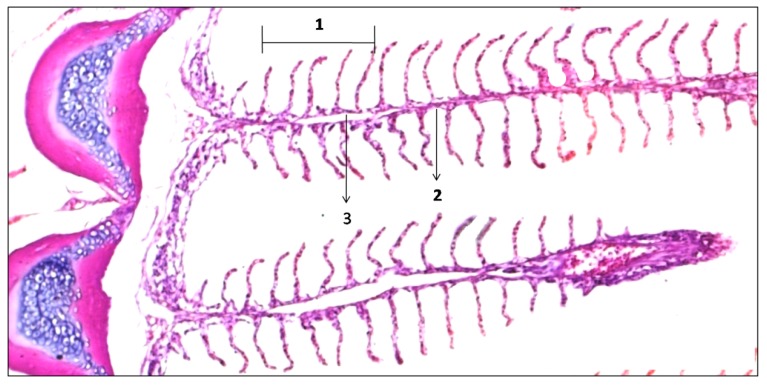
Normal gill filaments of *C. undecimalis*: 1: secundary lamella; 2: filament epithelium; 3: venous sinus (HE staining, 100× magnification).

The fish used as control showed a normal pattern of gill filaments (Figure 2); however, two stage I changes have been noted. Changes in stage I are considered mild and can be reversible with the improvement of environmental conditions, a fact evidenced in the research. Other researchers have also verified the reversibility of lesions in fish, during a period of 30 days [18,19].

The classification of histological alterations in the gills of fish collected in the Jansen Lagoon, is shown in Table 2.

**Table 2:** Classification of histological alterations of the gills of fish collected in the Jansen Lagoon, São Luís, Maranhão State, Brazil.

Stage I	Stage II
Lifting of the respiratory epithelium	Hemorrhage and rupture of the lamellar epithelium
Congestion of blood vessels	Aneurysm
Lamellar Disorganization	--
Hyperplasia of the lamellar epithelium	--
hypertrophy of the respiratory epithelium	--
Incomplete and complete fusion of several lamellae Parasite	--

The histological alteration index (HAI) for the gills of the species *Sardinella* sp and *C. undecimalis* varied from 4 to 25 and from 4 to 15, respectively, with a mean of 31.8 for *Sardinella* sp and 22.2 for *C. undecimalis*. The average HAI value in both species corresponds to category 21–50, *i.e.,* the gill tissue injuries of individuals sampled range from moderate to severe.

### 3.3. Gill Alterations

**Figure 3:**
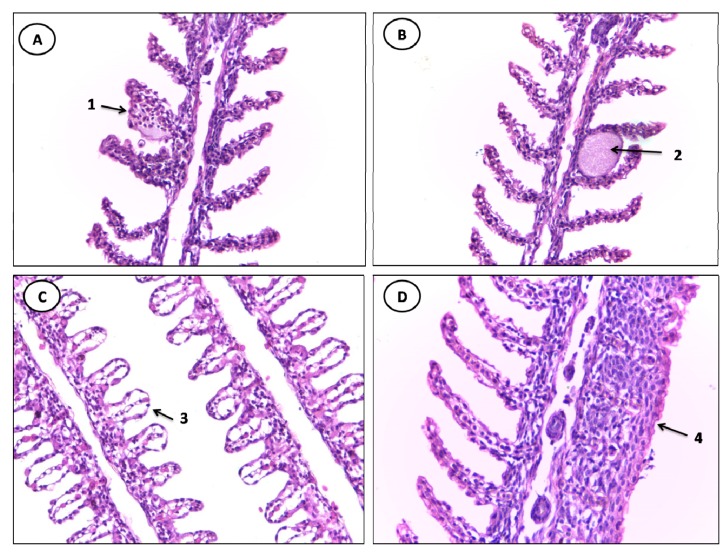
Histological alterations in the gills of *Sardinella* sp. (**A**) 1: aneurysm. (**B**) 2: parasitic cyst. (**C**) 3: lifting of the respiratory epithelium. (**D**) 4: complete fusion of several lamellae (HE staining, 400× magnification).

**Figure 4:**
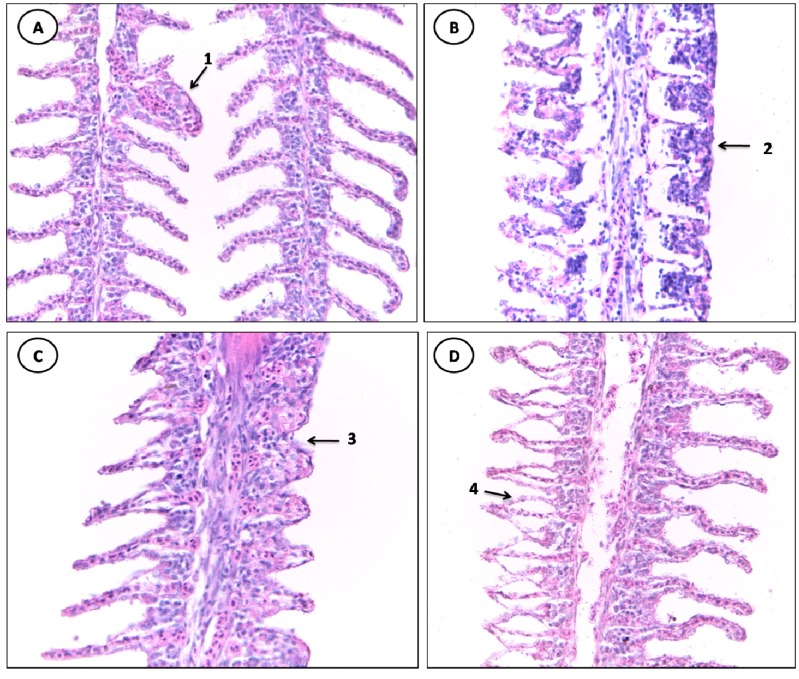
Histological alterations in the gills of *C. undecimalis*. (**A**) 1: aneurysm. (**B**) 2: complete fusion of several lamellae. (**C**) 3: incomplete fusion of several lamellae. (**D**) 4: lifting of the respiratory epithelium (HE staining, 400× magnification).

The gill tissue of specimens of *C. undecimalis* and *Sardinella* sp from the Jansen Lagoon showed the following histological alterations: lifting of the respiratory epithelium, hyperplasia of the lamellar epithelium, incomplete and complete fusion melting of several lamellae, lamellar disorganization, congestion of blood vessels, parasite, aneurysm, hypertrophy of the respiratory epithelium and hemorrhage and rupture of the lamellar epithelium (Table 3, Figure 3 and Figure 4).

**Table 3:** Frequency of histological alterations in the gills relating to fish species from the Jansen Lagoon, São Luís, Maranhão State, Brazil.

Alterations	Species		Total
	*Sardinella* sp	*Centropomus undecimalis*	
Lifting of the respiratory epithelium	18/18 (100%)	11/11 (100%)	29/29 (100%)
Hyperplasia of the lamellar epithelium	9/18 (50%)	10/11 (90.9%)	19/29 (65.5%)
Incomplete and complete fusion of several lamellae	16/18 (88.8%)	10/11 (90.9%)	26/29 (89.6%)
Lamellar disorganization	18/18 (100%)	10/11 (90.9%)	28/29 (96.5%)
Congestion of blood vessels	8/18 (44.4%)	2/11 (18.1%)	10/29 (34.4%)
Aneurysm	11/18 (61.1%)	2/11 (18.1%)	13/29 (44.8%)
Hypertrophy of the respiratory epithelium	11/18 (61.1%)	2/11 (18.1%)	13/29 (44.8%)
Hemorrhage and rupture of the lamellar epithelium	3/18 (16.6%)	0/11 (0%)	3/29 (10.3%)
Parasite	2/18 (11.1%)	0/11 (0%)	2/29 (6.9%)

From changes in stage I, the lifting of the respiratory epithelium was the most frequent lesion observed in all analyzed gills of samples of both species sampled, followed by hyperplasia of the lamellar epithelium and incomplete fusion of several lamellae with greater frequency in specimens of *C. undecimalis*.

The lifting of the respiratory epithelium is one of the earliest injuries found in fish; it is characterized by displacement of the lining epithelium of the secondary lamellae, in which the formation of a space called edema occurs, this being related to the presence of chemical contaminants, reduction of the gills’ surface, in addition to jeopardizing the gas exchange process [20]. Hyperplasia leads to the proliferation of adjacent lamellae cells, reducing the inter-lamellar space, which may cause a fusion of lamellae [3,21].

The gill lamellae of individuals sampled also showed stage II vascular changes considered as aneurysm, with a higher percentage in specimens of *Sardinella* sp. The aneurysm is characterized by the accumulation of blood in the secondary lamellae, caused by the rupture of pillar cells, thus increasing the blood flow, causing bleeding [22]. Similar results were obtained by Winkaler *et al.*, [20] who noted several changes, such as lamellar aneurysm and lifting of the lamellar epithelium, in gills of lambari (*Astyanax jacuhiensis*) from streams polluted by domestic sewage in Londrina, Paraná State, Brazil.

Meletti *et al.* [23], also found most changes in the gills of *Serrapinnus notomelas* and *Danio rerio*. The authors used histological injuries in gills, liver, and kidney as a tool in assessing the environmental degradation in the Mogi-Guaçu river basin, São Paulo State, Brazil.

The injuries found can be the gill’s defense mechanism against pollutants, and this can be associated with the discharge of domestic effluents, the eutrophication process and the presence of specific contaminants at the site. Rojas *et al.* [14], when analyzing the physico-chemical and nutritional characteristics of the Jansen Lagoon water, found that results showed the site may be under a process of eutrophication due to high concentrations of total phosphate, as well as contaminated, for the authors found a high concentration of dissolved aluminum.

Cantanhêde *et al.*, [17] in studies on *C. undecimalis* and Pereira *et al.* [24], with *Oreochromis niloticus* collected in the Jansen Lagoon found significant gill injuries related to the presence of untreated domestic effluents discharged into the lagoon environment.

Histopathology can be a sensitive tool for the detection of effects produced by toxic agents present in the lagoon, as it translates the lesion integrated due to the duration and intensity of exposure occurred and adaptive capacity of a certain tissue.

Several studies have shown that fish tissue alterations are reliable and efficient tools to detect and monitor environments that are influenced by anthropogenic activities [25,26,27,28,29], as well as to foresee potential environmental risks [30,31].

## 4. Conclusions

The high concentrations of thermotolerant coliforms in the analyzed water samples indicate the dumping of domestic effluents into the lagoon’s body of water compromising the site’s environmental quality.

The gill histological alterations detected were of first and second stage, being caused by environmental contamination by non-specific xenobiotics present at the site. These lesions, depending on the intensity, may be reversible when the presence of the xenobiotic ceases, therefore, gill lesions in fish from the Jansen Lagoon are biomarkers for assessing the water quality and assisting with environmental monitoring programs with the management bodies.
